# Photocrosslinkable Sericin Hydrogel Injected into the Anterior Chamber of Mice with Chronic Ocular Hypertension Efficacy, Medication Sensitivity, and Material Safety

**DOI:** 10.3390/bioengineering11060607

**Published:** 2024-06-13

**Authors:** Li Liao, Wenxiang Zhu, Hairong Liu, Ping Wu, Xinyue Zhang, Xiaoyu Zhou, Jiahao Xu, Yang Zhao, Xuanchu Duan

**Affiliations:** 1 Aier Academy of Ophthalmology, Central South University, Changsha 410015, China; 18374856298@163.com (L.L.); wuping3183@gmail.com (P.W.); 2 Aier Glaucoma Institute, Changsha Aier Eye Hospital, Changsha 410015, China; zhangxinyue3@aierchina.com (X.Z.); zhouxiaoyu2@aierchina.com (X.Z.); jiahao_xuu@163.com (J.X.); 3 College of Materials Science and Engineering, Hunan University, Changsha 410082, China; wenxiang@hnu.edu.cn (W.Z.); liuhairong@hnu.edu.cn (H.L.)

**Keywords:** sericin, photocrosslinkable, hydrogel, intraocular pressure, ocular hypertension model

## Abstract

(1) Background: A rise in intraocular pressure (IOP) and decreased retinal ganglion cells are frequent indicators of effective modeling of chronic ocular hypertension in mice. In this study, the sensitivity of the mouse model to pharmaceutical therapy to reduce intraocular tension was assessed, the model’s safety was confirmed using a cytotoxicity test, and the success rate of the mouse model of ocular hypertension was assessed by assessing alterations in IOP and neurons in the ganglion cell layer. (2) Methods: A mouse model of chronic ocular hypertension was produced in this study by employing photocrosslinkable sericin hydrogel injection and LED lamp irradiation. The eyes of 25 C57BL/6 male mice were subjected to 405 nm UV light from the front for 2 min after being injected with 5 μL of sericin hydrogel in the anterior chamber of the left eye. IOP in the mice was measured daily, and IOP rises greater than 5 mmHg were considered intraocular hypertension. When the IOP was lowered, the intervention was repeated once, but the interval between treatments was at least 2 weeks. The right eyes were not treated with anything as a normal control group. Mice eyeballs were stained with HE, Ni-type, and immunofluorescence to assess the model’s efficacy. Two common drugs (tafluprost eye drops and timolol eye drops) were provided for one week after four weeks of stable IOP, and IOP changes were assessed to determine the drug sensitivity of the mouse model of chronic ocular hypertension. Furthermore, CellTiter 96^®^ AQueous One Solution Cell Proliferation Assay (MTS) was utilized to investigate the safety of the ocular hypertension model by evaluating the deleterious effects of photocrosslinkable sericin hydrogel on cells. (3) Results: Before injection, the basal IOP was (9.42 ± 1.28) mmHg (1 kPa = 7.5 mmHg) in the experimental group and (9.08 ± 1.21) in the control group. After injection, cataract occurred in one eye, corneal edema in one eye, endophthalmitis in one eye, iris incarceration in one eye, and eyeball atrophy in one eye. Five mice with complications were excluded from the experiment, and twenty mice were left. Four weeks after injection, the IOP of the experimental group was maintained at (19.7 ± 4.52) mmHg, and that of the control group was maintained at (9.92 ± 1.55) mmHg, and the difference between the two groups was statistically significant (*p* < 0.05). Before the intervention, the IOP in the experimental group was (21.7 ± 3.31) mmHg in the high IOP control group, (20.33 ± 2.00) mmHg in the tafluprost eye drops group, and (20.67 ± 3.12) mmHg in the timolol maleate eye drops group. The IOP after the intervention was (23.2 ± 1.03) mmHg, (12.7 ± 2.11) mmHg, and (10.4 ± 1.43) mmHg, respectively. Before and after the intervention, there were no significant differences in the high-IOP control group (*p* > 0.05), there were statistically significant differences in the timolol eye drops group (*p* < 0.05), and there were statistically significant differences in the tafluprost eye drops group (*p* < 0.05). One week after drug withdrawal, there was no significant difference in IOP among the three groups (*p* > 0.05). In the high-IOP group, the protein (sericin hydrogel) showed a short strips or fragmented structure in the anterior chamber, accompanied by a large number of macrophages and a small number of plasma cells. The shape of the chamber angle was normal in the blank control group. The number of retinal ganglion cells decreased significantly 8 weeks after injection of sericin hydrogel into the anterior chamber, and the difference was statistically significant compared with the blank control group (*p* < 0.05). After the cells were treated with photocrosslinkable sericin hydrogel, there was no significant difference in the data of the CellTiter 96^®^ assay kit of MTS compared with the blank control group (*p* > 0.05). (4) Conclusions: A mouse model of chronic intraocular hypertension can be established successfully by injecting sericin in the anterior chamber and irradiating with ultraviolet light. The model can simulate the structural and functional changes of glaucoma and can effectively reduce IOP after the action of most antihypertensive drugs, and it is highly sensitive to drugs. Sericin has no obvious toxic effect on cells and has high safety.

## 1. Introduction

Glaucoma is the first irreversible cause of blindness in the world, with an estimated 76 million glaucoma patients worldwide in 2020 and an estimated 111.8 million by 2040 [1]. IOP is a clear risk factor for disease progression. Apoptosis and axonal degeneration of retinal ganglion cells (RGCs) are the main pathological bases of high IOP. It may eventually lead to blindness. At present, the only effective clinical treatment for glaucoma is to reduce IOP through drugs or surgery to prevent vision loss, but it cannot prevent the impact of retinal ganglion cell loss on visual function [2]. Glaucoma animal models have greatly improved our understanding of the causes and progression of glaucoma and promoted the development of antiglaucoma drugs and the improvement of treatment methods while searching for the pathogenesis of its risk factors. Animal models of different species, such as monkeys, dogs, rabbits, rats, and mice, have been applied in glaucoma-related research, among which the rodent model is the most widely developed and used [3]. Although these models have simulated the pathogenesis of human glaucoma well in some aspects, there are still shortcomings. How to construct an animal model consistent with the clinical manifestations and human pathogenesis based on the clinical characteristics of glaucoma is still the focus and difficulty of current research.

At present, various methods have been tried to establish an animal model of chronic ocular hypertension (COH) that reflects the basic characteristics of glaucoma. Generally, COH models can be divided into IOP-dependent and non-IOP-dependent models according to the pathogenesis. IOP dependence models include anterior chamber injection of microspheres [4], anterior chamber injection of silicone oil [5], laser photocoagulation model [6], suprascleral vein hypertonic saline injection model [7], suprascleral vein separation model [8], transgenic model [9], steroid drug application [10], optic nerve mechanical injury model [11], etc. The non-intraocular-pressure-dependent type includes the optic nerve mechanical injury model [11], retinal ischemia/reperfusion injury model [12], excitatory ganglion cell injury model [13], etc. Although these methods can mimic glaucomatous optic neuropathy caused by chronic IOP to some extent, most methods are limited to IOP elevation and RGC loss. The response to antihypertensive drugs and the safety of modeling methods are often neglected. The unilateral evaluation method reduces the reliability of the model, and the lack of safety considerations brings hidden dangers to its application. Therefore, this study evaluated the damage to visual function, the response to two common antihypertensive drugs (timolol maleate eye drops and tafluprost eye drops), and the effect of hydrogel injection on the structure of the anterior chamber and cells to more comprehensively evaluate the COH model and provide a basis for glaucoma basic research.

## 2. Materials and Methods

### 2.1. Materials

SerMA: Lithium phenyl-2,4,6-trimethylbenzoylphosphinate (LAP) (Shanghai Yinchang New Material Co., Ltd., Shanghai, China) (5 mg/mL) and SerMA (5 mg/mL) were combined in phosphate-buffered saline (PBS) (0.01 M PBS (powder, pH 7.2–7.4)) (Bioss Antibodies, Bioss Inc., 300 Tradecenter Dr, Ste 4610, Woburn, MA, USA) to create the precursor SerMA hydrogel solutions. By exposing the precursor SerMA hydrogel solutions to blue light at 405 nm, the hydrogels were subsequently transformed into SerMA. Instrument: TonoLab Rebound tonometer (Tonolab; Tiolat Oy, Helsinki, Finland); ophthalmic surgical microscope system (OMS-800 Standard; TOPCON, Tokyo, Japan); LED light (UV 405 nm-20 W, Guangzhou Guanghe Tongsheng Technology Co., Ltd., Guangzhou, China); insulin syringe (0.3 mm × 8 mm 1 mL/40 I.U. U-40, Shanghai, China). Device: Small animal imaging integrated platform, phoenix micron IV; drugs: IOP-lowering drugs: Tafluprost eye drops (0.0015% tafluprost eye drops, Santen Pharmaceutical Co., Ltd., Shiga Plant, Shiga, Japan); timolol maleate eye drops (0.5% timolol maleate eye drops, Wujingyaoye, Wuhan, China); mydriatic: Tropicamide phenylephrine eye drops, 1 mL: Topicamide 5 mg and deoxyadrenalin hydrochloride 5 mg, Santen Pharmaceutical Co., Ltd., Shiga Plant, Shiga, Japan; anesthetic: Intraperitoneal injection, 1% sodium pentobarbital (Pelltobarbitalum Natricum, Merck, Darmstadt, Germany); anti-inflammatory drug: Levofloxacin hydrochloride eye drops (levofloxacin eye drops, 24.4 mg:5 mL, Santen Pharmaceutical Co., Ltd., Shiga Plant, Shiga, Japan); chlortetracycline hydrochloride eye ointment (chlortetracycline hydrochloride eye ointment, Shuangji, Beijing, China). SerMA was synthesized with reference to previous reports in the literature [14,15].

### 2.2. Animal Welfare and Anesthesia

This study was approved by the Hunan SJA Laboratory Animal Co., Ltd Ethics Committee and follows the guidelines of the Society for Research in Vision and Ophthalmology (ARVO). All animals were purchased from Hunan SJA Laboratory Animal Co., Ltd., Changsha, China. The mice were 25 C57BL/6 males, 4 weeks old, weighing about 20 g, and were kept in a standard cage where water and food were freely available and kept in a 12 h light-dark cycle under controlled temperature (22 °C) and relative humidity (55%). Two weeks before the experiment, IOP was measured with a TonoLab tonometer while awake to acclimate to the procedure and minimize mistakes. A week before the trial, the baseline IOP was measured. Under general anesthesia (1% pentobarbital sodium, 50 mg/kg, intraperitoneal injection) and local eye drops (0.4% obucaine hydrochloride eye drops, intraoperative eye), anterior chamber photocrosslinkable sericin hydrogel injection was carried out. During the entire operation and anesthesia, the environment was strictly disinfected and the temperature was controlled using a heating pad and the mice were placed in an oxygen-enriched 25% environment after surgery.

### 2.3. High Intraocular Pressure Induced in the Injection Process

**Figure 1:**
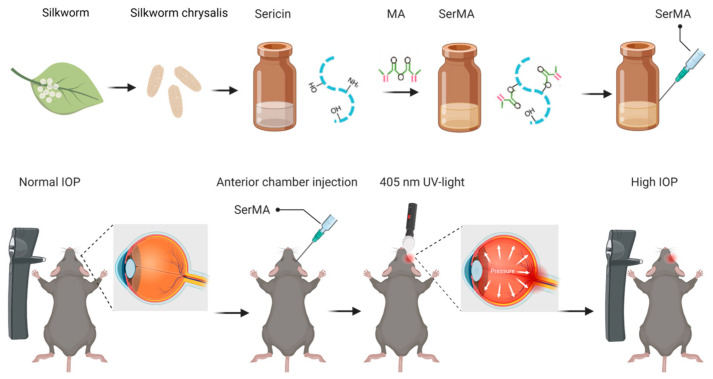
SerMA hydrogel synthesis process and high-IOP modeling process diagram (Red represents the carbon-carbon double bond).

In this study, a mouse model of intraocular hypertension was established by anterior chamber photocrosslinkable sericin hydrogel injection (Figure 1). Before the operation, 0.5% levofloxacin eye drops were applied onto the operative eye to prevent infection, and compound tropicamide eye drops were added again at an interval of 5 min to dilate the pupil. The mice were anesthetized with 1% sodium pentobarbital injected peritoneally, and the corneal surface was anesthetized with 0.4% obucaine hydrochloride eye drops. Then, 5 μL sericin was injected into the anterior chamber through the temporal-corneosclera margin with an insulin syringe (31 G × 5 mm), and then the center of the cornea was covered with a light mask, and the eyeball was irradiated with 405 nm ultraviolet light from the front for 2 min. After the hydrogel material was solidified in the peripheral anterior chamber, 0.5% levofloxacin eye drops and aureomycin eye ointment were applied to the eyes to prevent infection. IOP was measured daily, and if the IOP remained elevated by more than 5 mmHg, it was defined as high IOP. If IOP is reduced, the intervention was repeated once more, but at least 2 weeks later. The right eye was the experimental group and the left eye was the control group.

### 2.4. Clinical Signs and Intraocular Pressure Measurement

Clinical signs such as ocular congestion, corneal changes, infection, and intraocular inflammation were assessed weekly. IOP was measured with a TonoLab rebound tonometer and 3 measurements were repeated with the TonoLab (which averages the values of 6 consecutive measurements). To mitigate deviations caused by circadian rhythms, measurements are strictly taken between 10 a.m. and 2 p.m. All IOP measurements were performed by the same operator.

### 2.5. Histopathological Observation

Four weeks after anterior chamber injection, the IOP remained steady, and 15 mice with high IOP were kept under observation until nine weeks had passed since injection. They were then randomly separated into three groups for drug sensitivity testing. Both eyes (including the optic nerve) were swiftly removed and fixed in 40 g/L paraformaldehyde after the remaining mice were euthanized. The tissue was embedded in gradient alcohol dehydration and sliced with a thickness of 3 μm parallel to the ocular axis 24 h later. The optic nerve was cut transversely, dewaxed, and dried with water, and the baking sheet was waxed. The following procedures were then carried out: HE staining, toluidine blue staining, and immunofluorescence staining.

### 2.6. HE Staining

HE staining was performed as previously described. The slices undergoing HE staining were retinal slices. Slices were placed in xylene I for 20 min, xylene II for 20 min, anhydrous ethanol I for 5 min, anhydrous ethanol II for 5 min, and 75% alcohol for 5 min before being washed with tap water. Hematoxylin was then used to stain the slices for 3 to 5 min before being differentiated in hydrochloric acid solution and then turned blue in ammonia solution. Sections were stained for 5 min in an eosin solution after being sequentially dehydrated with 85% and 95% gradient alcohol. Slices were successively placed in anhydrous ethanol I, anhydrous ethanol II, anhydrous ethanol III, dimethyl, xylene II, and neutral gum sealing tablets for 5 min each, followed by microscopy, image collection, and analysis.

### 2.7. Toluidine Blue Staining

Toluidine blue staining was performed as described previously. The slides undergoing toluidine blue staining were optic nerve slices. The slices was placed in xylene I for 20 min, xylene II for 20 min, anhydrous ethanol I for 5 min, anhydrous ethanol II for 5 min, and 75% alcohol for 5 min and washed with tap water. The slices were immersed in the dye for 5 min, washed with water, and slightly differentiated with 1% glacial acetic acid, and the reaction was terminated by washing with tap water. The degree of differentiation was controlled under the microscope, and the slices were dried in the oven after washing with tap water. Put the slices into clean xylene for 5min and seal with neutral gum. Microscopy, image acquisition, and analysis were then carried out.

### 2.8. Immunofluorescent Staining

Immunofluorescence staining was performed as previously described. First, paraffin sections were dewaxed in water, as above. The tissue sections were then placed in a repair box filled with EDTA antigen repair buffer (PH8.0) for antigen repair in a microwave oven. After the sections were slightly dried, a circle was drawn around the tissue with a histochemical pen (to prevent the antibody from flowing away), then a self-fluorescence quenching agent was added within the circle for 5 min and rinsed with water for 10 min. BSA was added to the circle and incubated for 30 min. The sealing solution was gently shaken off, PBS was added to the slices with a certain proportion of primary antibody, and the slices were placed flat in a wet box at 4 °C for overnight incubation. The slides were placed in PBS (PH7.4) and washed by shaking on a decolorizing shaker 3 times, 5 min each time. After the slices were slightly dried, the tissue covered by the second antibody of the corresponding species of the first antibody was added to the ring and incubated at room temperature for 50 min away from light. The slides were placed in PBS (PH7.4) and washed by shaking on the decolorizing shaker 3 times, 5 min each time. After the slices were slightly dried, DAPI dye was added to the circle and incubated for 10 min at room temperature away from light. The slides were placed in PBS (PH7.4) and washed by shaking on the decolorizing shaker 3 times, 5 min each time. The slices were slightly dried and sealed with antifluorescence quenching tablets. The sections were observed under a fluorescence microscope and the images were collected.

Complete eye and optic nerve sections of the experimental group and control group were selected. ImageJ (ImageJ 1.53 t) image processing software was used to analyze the changes in the number of GCL nuclei in the two groups in retinal HE staining and the visual changes in the morphology of retinal nerve fibers in toluidine blue staining. The angle morphologies of the experimental group and the control group were observed with Case Viewer.

### 2.9. Effects of Two IOP Drugs on the COH Model

Tafluprost eye drops are a new PGF2α derivative, and the main mechanism of reducing IOP is to promote the outflow of aqueous humor through the uveal sclera, thereby reducing IOP. Since its introduction as a selective PG receptor agonist in 2008, preliminary studies have shown that preserved and preservative-free preparations of tafluprost have proven to be effective and safe in the treatment of glaucoma and ocular hypertension [16]. Timolol maleate eye drops, as a classic antiglaucoma drug, are β-blockers whose main mechanism of action is to reduce IOP by reducing the secretion of aqueous humor by the ciliary body [17]. The two IOP medications mentioned above, which encourage aqueous humor outflow and limit aqueous humor secretion, were utilized in this investigation to assess the COH model’s drug sensitivity. Four weeks after the COH model’s IOP had stabilized, with the IOP controlled at around 25 mmHg, the COH model was randomly divided into the control group (saline group) and the experimental group (tafluprost eye drops and timolol maleate eye drops), with five eyes in each group. The IOP changes were observed 2 h before and after 0 h, 2 h, 4 h, 6 h, 8 h, 24 h, 2 to 7 days of drops from the first day. Each dose is about 15 μL.

### 2.10. Toxic Effect

Human Tenon capsule fibroblasts (HTFs) were isolated from individuals undergoing strabismus surgery who had no history of conjunctival disease or use of topical ocular medication as previously described [18]. The HTFs were cultured in Dulbecco’s modified Eagle medium supplemented with 10% (*v*/*v*) fetal bovine serum (Hyclone Laboratories, Logan, UT, USA) and 1% streptomycin–penicillin (Gibco, Thermo Fisher Scientific, Waltham, MA, USA) in a humidified atmosphere at 37 °C with 5% CO_2_. HTFs cultured in the above medium were a complete medium group (CM group). Passages 4–6 were used for further experiments. Prior to each experiment, the cells were allowed to reach a sub-confluent state (~80% confluence); after which, they were cultured in a serum-free medium for 24 h.

As previously mentioned, cytotoxicity was determined using MTS. HTFs were first inoculated in 96-well plates with 100 μL HTFs per well. Then, 20 μL hydrogel was added to the culture hole and cultured for 1, 2, 4, and 7 days, respectively. The MTS reagent was removed and dissolved at room temperature or 37 °C. Then, 10 μL of MTS reagent was added to 96-well plates and cultured for 1, 2, 4, and 7 days. Then, the 96-well plates were put back into the incubator and incubated at 37 °C for 2 h. Finally, OD values were measured at 490 nm using an enzyme-labeled instrument. The OD value measured at 490 nm is directly proportional to the number and viability of living cells in the culture.

### 2.11. Statistical Analysis

Data were recorded in an Excel database using SPSS software (IBM SPSS Statistics Version 19.0; IBM, Armonk, NY, USA) and statistical analysis was performed. The difference in IOP in each group before and after the intervention was statistically determined by a Mann–Whitney U test (Wilcoxon rank sum test). The difference in IOP between groups after the intervention was statistically determined by the Welch one-way ANOVA test and multiple hypothesis test (Tukey HSD post hoc tests). All data are expressed as mean ± standard deviation. *p* < 0.05 (represented by *) was statistically significant.

## 3. Results

### 3.1. The Impact of Medication on Lowering Intraocular Pressure

**Figure 2:**
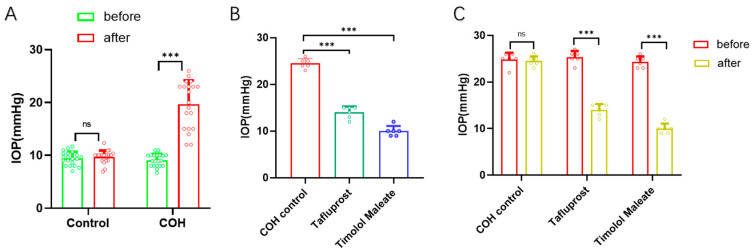
(**A**) Changes in IOP in the COH group and the control group before and after injection of photocrosslinkable sericin hydrogel into the anterior chamber (the COH group received anterior chamber injections, whereas the control group did not) (the sample size, *n* = 20); (**B**) Differences in IOP between groups before and after different IOP-lowering drug interventions (the sample size, *n* = 6); (**C**) Changes in IOP in each group before and after intervention with different IOP-lowering drugs (the sample size, *n* = 6). (^ns^ *p* > 0.05, *** *p* < 0.001).

From Figure 2, we can see the changes in IOP in the COH group and the control group before and after the intervention (Figure 2A). The IOP of the COH group was increased after anterior chamber photoinduced sericin hydrogel injection, which was statistically significant compared with that before injection (*p* < 0.05). The control group was not treated, and the difference was not statistically significant (*p* > 0.05). After the high IOP was stable for 4 weeks, the mice were randomly divided into three groups, which were given normal saline (control group), tafluprost eye drops, and timolol maleate eye drops, respectively, and the changes in IOP in each group were observed. The IOP of each group before and after the intervention is shown in Figure 2C. Compared with IOP before the intervention, the IOP of the tafluprost eye drops group and the timolol maleate eye drops group decreased significantly after intervention, and the differences are statistically significant (*p* < 0.05). There was no significant difference in IOP in the control group (*p* > 0.05). The changes in IOP in each group after intervention are shown in Figure 2B. Compared with the control group, there were statistically significant differences in IOP between the tafluprost eye drops group and the timolol maleate eye drops group (*p* < 0.05), and there were also statistically significant differences in IOP between the two experimental groups (*p* < 0.05).

**Figure 3:**
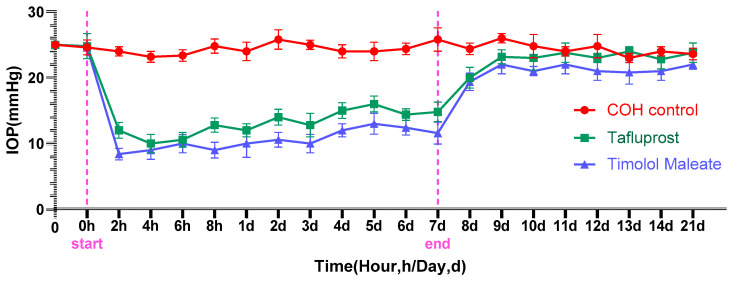
The trend of IOP in each group before and after intervention with different IOP-lowering drugs.

Changes in IOP before and after administering sericin hydrogel in the three experimental groups are shown in Figure 3, starting from the “start” time point and stopping at the “end” time point. During the whole intervention process, the IOP of the control group (drops of normal saline) was basically maintained at 22 mmHg. However, 2 h before administration, the IOP of the three groups was about 22 mmHg and, 2 h after administration, the IOP of the experimental group dropped to the lowest level, about 10 mmHg, and then rose slightly but the trend was stable. Between them, the decrease in the timolol maleate eye drops group was always greater than that of the tafluprost eye drops group. IOP in the two experimental groups rose the fastest within 2 days after drug withdrawal and slowly and steadily within 5 to 7 days and tended to the level before the experiment but could not reach the high IOP measured before the experiment.

### 3.2. Clinical Characteristic Change

**Figure 4:**
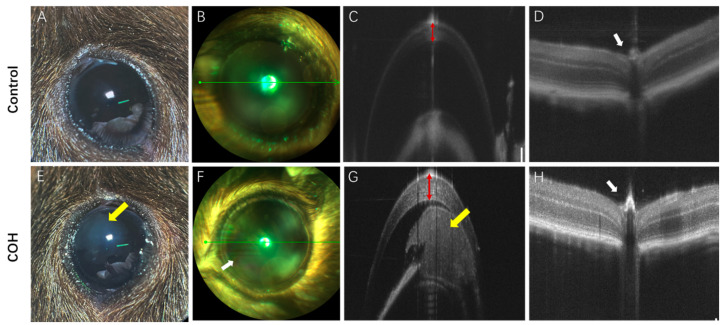
Evaluation of the anterior chamber and retina in vivo after sericin hydrogel administration. (**A**,**B**) Anterior segment status of the control group; (**E**) the white nebulous jelly-like substance in the front chamber indicated by the yellow arrow is sericin hydrogel solidified after light induction; (**F**) the white arrow indicates bleeding in the anterior chamber; (**C**,**G**) the red arrow shows corneal thickness; (**G**) the yellow arrow indicates a photocrosslinkable sericin hydrogel; (**D**,**H**) the white arrow indicates the mouse optic disc.

**Figure 5:**
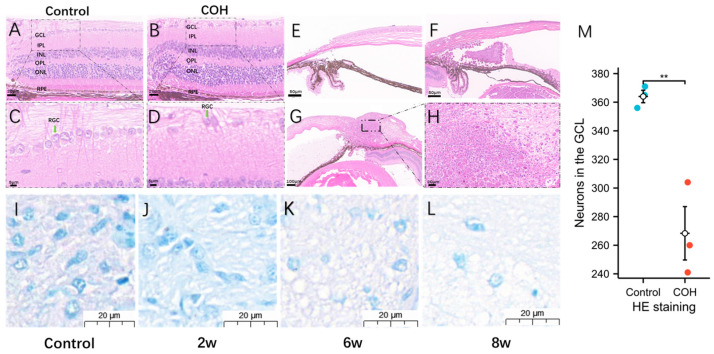
Anatomical changes in the retina, cornea, and optic nerve after sericin-hydrogel-induced COH. (**A**–**H**) is the result of HE staining. (**A**,**C**) Retinal layers and neurons in the GCL of control group; (**B**,**D**) Retinal layers and neurons in the GCL of COH group; (**E**) Anterior chamber angle morphology of control group; (**F**) Anterior chamber angle morphology of COH group; (**G**,**H**) Corneal edema in COH group; (**I**–**L**) Optic nerve toluidine blue staining was performed and (**I**–**L**) respectively present before, 2 w, 6 w, and 8 w after injection of photocrosslinkable sericin hydrogel into the anterior chamber (scale bar = 20 μm); (**M**) Quantitative analysis of neurons in the GCL was performed in HE staining (the sample size, *n* = 3) (** *p* < 0.01).

As can be seen from Figure 4, mice had good tolerance to photocrosslinkable sericin hydrogel, and milky white sericin (Figure 4E) could be seen in the anterior chamber, but the diopter medium of both the IOP group and the control group could remain clear, and the lens was transparent under the light mirror, while the retina and optic disc under the fundus could be seen (Figure 4B,D,F,H). Although the incidence of corneal edema (Figure 5G,H) of different degrees is higher after early injection, or there is anterior chamber bleeding (Figure 4F), the daily application of levofloxacin drops and aureomycin eye ointment in the first week after injection can gradually alleviate corneal edema and the corresponding inflammatory reaction. In addition, the results of the five mice were excluded from the analysis with iris incarceration and cataract in two operative eyes, unimproved corneal edema leading to corneal endothelial decompensation in one operative eye, and endophthalmitis with atrophy in the other two operative eyes.

### 3.3. Morphological and Degenerative Changes in Ganglia after Increased Intraocular Pressure

After injection in the anterior chamber, sericin always filled the peripheral anterior chamber (Figure 4E,G and Figure 5F). The results were consistent with those of HE staining. Despite the degradation of sericin, it still retained its state and function until the end of the experiment. The pathological anatomy of the eyeball of the mice with high IOP showed ciliary body edema, short strips or fragments of protein-like structures in the anterior and/or posterior chambers, accompanied by infiltration of macrophages, plasma cells, and inflammatory cells. In HE staining, compared with the control group (Figure 5A,C), the number of neurons in the ganglion cell layer (GCL) in the COH group (Figure 5B,D) was significantly reduced (Figure 5C,D), and it could be seen that the number of nuclei gradually decreased with time in the optic nerve type staining (Figure 5I–L). Quantitative analysis of neurons in the GCL showed that there were fewer neurons in the GCL in the retina of mice in the COH group than in the control group, and the difference was statistically significant (*p* < 0.05) (Figure 5M).

### 3.4. The Hydrogel’s Non-Cytotoxicity for HTFs Was Demonstrated by the MTS Assay

**Figure 6:**
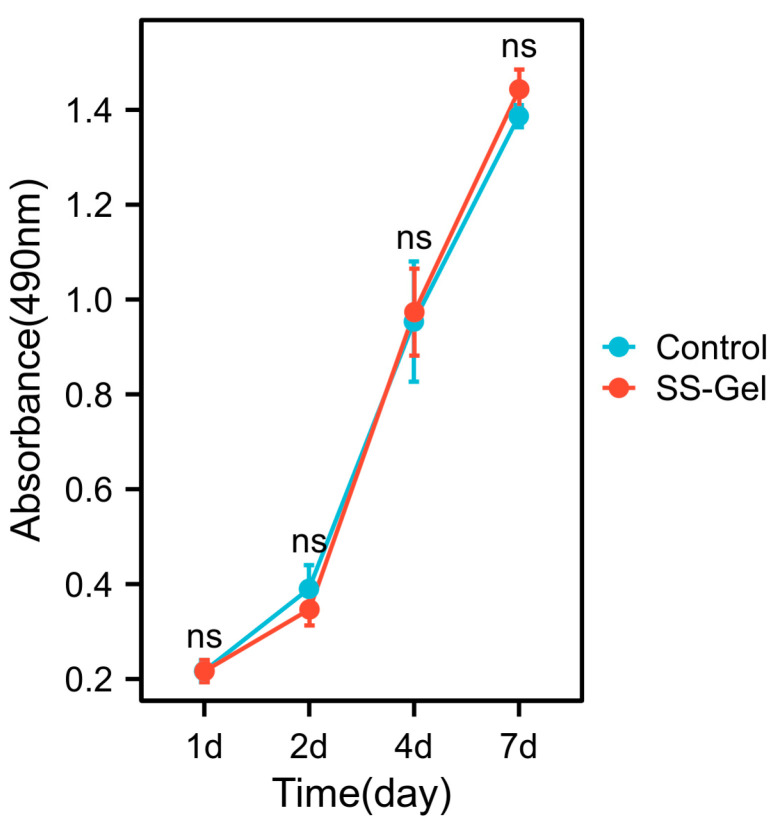
Effect of crosslinked hydrogels on the cytotoxicity of HTFs. Comparison of OD values at different time points between the control group and the crosslinked hydrogel co-culture group (the sample size, *n* = 9) (^ns^ *p* > 0.05).

MTS is used to ascertain whether hydrogels are cytotoxic. MTS is a homogeneous unlabeled cell viability assay that uses a light absorption assay as its foundation. It offers a quick and precise way of analyzing cell proliferation and toxicity. Living cells can convert the reagent into a very water-soluble methyl-formazan dye that can be detected at 490 nm. The quantity of methyl-formazan produced is inversely correlated with the quantity and health of living cells. The quantity and health of the cells in the hydrogel co-culture group and the control group were examined in this study. The OD values steadily increased as the culture time was extended. The hydrogel co-culture group’s OD values did not differ significantly from those of the control group, and there was no statistically significant variation between any of the time points (Figure 6). These findings show that the crosslinked hydrogels are highly cytocompatible and non-cytotoxic to HTFs.

## 4. Discussion

The development of the COH model offers a research foundation for the occurrence, progression, and treatment of glaucoma. In this research, we present a novel COH modeling approach that departs from the traditional COH modeling approach. Photocrosslinkable sericin hydrogel is injected into the anterior chamber to block the chamber’s corner, increasing IOP. Following successful modeling, various techniques were employed to assess the COH model’s efficacy, medication sensitivity, and injectable material safety. COH modeling is an interdisciplinary combination of ophthalmology and materials science, and it is hoped that a new COH model can be established by using the advantages of interdisciplinary disciplines to provide ideas for the development and improvement of glaucoma animal models and provide theoretical guidance and a basis for clinical research on glaucoma.

### 4.1. Analysis of the COH Model Created by Anterior Chamber Injection

In this study, a certain amount of sericin hydrogel was injected into the anterior chamber of C57BL/6 mice to slow down or block the outflow of aqueous fluid by increasing the outflow resistance or blocking the trabecular network, resulting in increased IOP, thus establishing the COH model. Previous studies have shown that the main materials used for anterior chamber injection are: Magnetic microbeads, compound carbomer, sodium hyaluronate, and polylactic-glycolic acid copolymer (PLGA) microspheres [9]. Kim et al. [19] found that anterior chamber injection of 0.3% carbomer solution is an effective and reproducible method to generate chronic intraocular pressure. In contrast, in this study, the IOP increased significantly in the first 1–2 days after a single injection and was maintained for an average of 8 weeks. IOP stabilized within 4 weeks after a single injection of photoinduction sericin hydrogel and decreased slightly at 4–8 weeks, but it was still in the range of high IOP (Figure 2 and Figure 3). For the mice whose IOP was not significantly increased after a single injection, the IOP could still be increased and maintained in the state of high IOP after repeated injections one week apart. Alba et al. [20] established a model of intraocular hypertension in chronic glaucoma by injecting 2 microliters of polylactic acid co-glycolic acid mammal fiber suspension (10% *w*/*v*) into the right eye of 45 Long Evans rats, observed for 6 months, and found that the RGC function of the injected eye was reduced. A decrease in toluidine-blue-positive nuclei may indicate a decrease in the number of certain types of glial cells in the optic nerve or a morphological change (Figure 5I–K). Toluidine blue can label the nuclei of astrocytes, microglia, oligodendrocytes, and oligodendrocyte progenitors. Astrocytes provide structural support and nutrients in the retina and are essential for maintaining retinal health. In the COH environment, astrocytes may become dysfunctional due to constant stress [21]. Microglia are the main immune cells of the retina and are sensitive to injury. Under COH conditions, microglia may change from a homeostatic state to an active state [22]. Oligodendrocytes provide myelin support for the axons of the optic nerve. Axon loss may result in reduced numbers or functional degradation of oligodendrocytes and their progenitors [23]. COH may induce the degeneration of these cell populations through various mechanisms, including oxidative stress, the release of inflammatory factors, and the deficiency of nutritional factors [24,25]. The decrease in the total number of toluidine-blue-positive nuclei indicates that the optic nerve tends to degenerate with the prolongation of intraocular hypertension.

### 4.2. The Mechanism of Causing COH by Photocrosslinkable Sericin Hydrogel into the Anterior Chamber

The first basis is the growth and/or remodeling of the load-bearing connective tissue of the optic nerve head (ONH), which leads to elevated IOP and damage to the RGCs. The second basis is progressive damage to adjacent axons through multiple pathways, such as oxidative stress, inflammatory response, and metabolic disorders, all of which may contribute to the death of RGCs. These results reveal the complexity and diversity of the pathogenesis of glaucoma [26,27]. Tribble et al. [28] injected 6–8 μL (1.6 × 10^6^ pieces/μL) of epoxy resin magnetic microspheres with a diameter of 4.5 μm into the anterior chamber of brown Norway rats once and blocked the corner of the chamber with hand-held magnet attraction. However, this method requires repeated injections, which carries a high risk of corneal decompensation and intraocular bleeding [29,30]. In addition, the presence of residual microbeads in the retina may affect the observation and counting of RGCs [30]. Yu et al. [31] injected 7 μL in situ crosslinked hydrogel premixed with carboxymethyl hyaluronic acid and polyethylene glycol diacrylate at a ratio of 4:1 in the anterior chamber of Sprague Dawley rats to increase the IOP, which effectively simulated the pathological changes occurring in human glaucoma. According to the study of Nadal-Nicolas et al., there are many ways to label retinal ganglion cells [32] and among the approximately 217,406 cells that make up the GCL (excluding endothelial cells), 10% are glial cells, 50% are non-RGC neurons, and the remaining 40% are RGCs [33]. We quantitatively expressed the GCL by HE staining to verify intraocular hypertension and demonstrated that anterior chamber injection of sericin hydrogel is of guiding significance in studying RGC-related structural and functional degeneration (Figure 5). At the same time, this study combined light induction and material properties to observe its degradation in the anterior chamber, it is simple to operate, and a single injection has a significant effect on increasing IOP.

Injection of the hydrogel can simulate both open-angle and closed-angle glaucoma, which is a novel method to study the mechanism of intraocular hypertension. Although the hydrogel will gradually absorb, it will block the angle of the chamber and affect the flow of fluid in the eye, thereby increasing the IOP, resulting in the death of retinal neurons and loss of vision.

### 4.3. Comparison of the Efficacy of the COH Model

There are many methods to cause COH, and the efficacies of various COH models differ. Zhang et al. [34] compared the isolation of the epcleral vein with the combination of 270° limbal blood vessel occlusion and found that it is prone to complications such as corneal decompensation and exophthalmos. Shruti et al. [35] established a COH mouse model by encoding the activity HTGF-β2C226,228S in a lentiviral gene vector to induce trabecular network dysfunction and increase outflow resistance of the atrial stream. IOP increased significantly 3 weeks after injection in the experimental group compared to the control eye, with a mean incremental change of 3.3 mmHg, which remained elevated for 7 weeks after injection. By contrast, the results of our study showed that the experimental group of mice stabilized at high IOP 4 weeks after injection and this lasted for more than 10 weeks, among which two mice were able to increase IOP again after repeated injection (Figure 2). Zahavi et al. [36] injected plastic microbeads into the anterior chamber of mice, and the results showed that the mean IOP at 4 weeks after a single injection was significantly higher than the baseline of the injected eye (14.5 ± 3.3 mmHg vs. 11.1 ± 2.5 mmHg, *p* = 0.003). The IOP did not increase in the control group (13.2 ± 2.9 mmHg vs. 12.2 ± 2.9 mmHg). Compared with the study of Zahavi et al., the IOP increased to a greater extent in this study (Figure 2 and Figure 3). Compared with the above COH model construction method, the photoinduction sericin hydrogel was injected into the anterior chamber of mice to create a chronic intraocular hypertension model. After LED UV irradiation, sericin hydrogel could solidify and then block the corner of the chamber and trabecular network, which was convenient for tracking the distribution and degradation, at the same time keeping the diopter interstitium clear. It will not accumulate in the pupil area and affect the fundus examination through the pupil (Figure 4). More importantly, the use of photoinduced sericin hydrogel requires fewer injections to achieve a sustained increase in IOP, which greatly improves the reliability of experimental results. The above comparison proves that anterior chamber injection of photoinduced sericin hydrogel is an interdisciplinary and novel method for constructing glaucoma models.

### 4.4. The Application of Hydrogels in Medicine

In recent years, hydrogels have been proven to have low toxicity, high water retention ability, multifunctional use, and excellent biocompatibility and have been applied in many biomedical research fields such as regenerative medicine therapy, systemic sclerosis, and rheumatoid arthritis [37,38]. Hydrogels have been widely studied in the fields of oncology, tissue engineering, ophthalmology, and biological detection [39]. Their applications in the field of ophthalmology include drug delivery systems for various eye diseases such as corneal neovasculature, glaucoma, and uveitis treatment, the treatment of ocular surface and lacrimal duct diseases, vitreous replacement, etc. [40,41]. Sericin hydrogel can be stored in liquid form at room temperature with closed light and can be cured after LED purple light induction. Therefore, we chose to induce COH with LED purple light irradiation after injecting it into the anterior chamber. Even with only one injection, the IOP rise in the model was stable and sustained, which will help reduce the possibility of infection and provide an adequate time window for studying the pathological changes of this chronic disease. This advances the application of crosslinked hydrogels in glaucoma.

### 4.5. Complications of the COH Model

In different literature reports, the increased IOP caused by microsphere injection and its effects on RGCs were also different. The increase in IOP from 27% to 118% for 8 weeks reduced RGCs by 50%, and the decrease in IOP from 40% to 61% only caused the loss of RGCs from 4.1% to 5.2%. The advantage of this model is relatively simple operation technology, but the disadvantage is that multiple injections may cause adverse reactions such as abnormal cornea and increased incidence of inflammation [4]. Different models of intraocular hypertension can have different effects on the ocular tissue. However, similar to the above findings, in addition to the decrease in retinal nerve fibrocytes, the COH model in this study showed different degrees of lesions, including ciliary body edema, proteinoid, macrophages, and plasma cell infiltration in the anterior and/or posterior chambers. The proteinoid is homogeneous (inflammatory exudate). These results indicate that our model effectively simulates the pathological changes that occur in human glaucoma and has the potential for further application in a series of studies of RGC-related degeneration in terms of structure and function by injecting sericin hydrogel into the anterior chamber.

### 4.6. Medication Sensitivity Analysis of the COH Model

After proper injection in the anterior chamber, the crosslinked hydrogel closed the corner of the chamber and blocked the path of outflow of aqueous humor. The COH model can be used for preclinical evaluation of the effectiveness of aqueous humor inhibitors. Our results showed that both timolol maleate eye drops, which inhibited the formation of aqueous humor, and tafluprost eye drops, which promoted the outflow of aqueous humor, had a good effect on reducing IOP induced by hydrogel COH, which is similar to the results of a Korean glaucoma study [42]. Interestingly, timolol maleate eye drops have a faster onset, stronger action, and relatively lower IOP at the end of action compared to tafluprost eye drops. The IOP of both groups decreased significantly 2 h after administration. Among them, tafluprost eye drops reached the lowest value 4 h after administration to the COH model, while timolol eye drops reached the lowest value 2 h after administration to the COH model (Figure 2 and Figure 3). We speculated that this might be related to the mechanism of action of the two eye drops. Considering that hydrogel induces intraocular hypertension by blocking the angle of the anterior chamber and trabecular reticulum, based on this result, we speculated that the mechanism of action of this sericin hydrogel in the COH model is more related to the mechanism of open-angle glaucoma, and certain evidence was also shown in the results of HE staining, which is similar to that of Qiang Yang et al. [43]. The results of this study showed that IOP rose rapidly 2 days after drug withdrawal, and then rose slowly and gradually until it stabilized at about 18 mmHg, which was slightly lower than that of the control group. Their main function is to increase IOP by blocking the angle of the chamber and changing the structure of the anterior chamber and ciliary body.

### 4.7. Material Safety Analysis of the COH Model

In the biosafety assessment of hydrogels, we have studied the effect of hydrogel injection on the structure of the anterior chamber and the toxic effect on HTFs [44]. The results showed that there was no significant change in the anterior segment of the COH group, and co-existing with crosslinked hydrogel had no significant effect on the apoptotic state of HTFs. It was also found that anterior chamber injection of crosslinked hydrogels had no significant toxic effect on the anterior chamber angular structure nor on the proliferation and apoptosis of HTFs, which allowed us to use hydrogels with more confidence in modeling (Figure 6).

## 5. Advantages and Limitations

In this study, sericin hydrogel was injected into the corner of the glaucoma animal model and then irradiated with LED ultraviolet light, so that the hydrogel was solidified in the corner of the anterior chamber, thus obstructing the outflow of aqueous humor and resulting in the increase in IOP. The advantages are as follows: (1) High stability: Compared with other methods, the blockage formed after hydrogel injection is more stable, can last for some time, and is more suitable for long-term observation and research. (2) Easy operation: Hydrogel injection is relatively simple, does not require complex instruments and technology, and can reduce the harm to animals. (3) Wide range of application: Hydrogel injection can be used in a variety of animal models, such as rats, rabbits, etc., with a wide range of applications. (4) Silk hydrogel can be gradually degraded and absorbed in the body, avoiding long-term implant safety risks in the body. This study encountered several limitations in exploring the ocular hypertension model, which may have implications for the interpretation and generalization of the findings: (1) Model choice: The animal model of intraocular hypertension we used may not be able to fully replicate all the complexities of elevated intraocular pressure in humans. Although this model is widely used in ophthalmology studies, it may not cover all clinical manifestations and pathological changes in glaucoma patients. (2) Species of experimental animals: Different animal species may have different physiological responses to increased intraocular pressure. Rodent models differ physiologically and behaviorally from humans, which may limit the general applicability of our findings. Therefore, we plan to introduce monkeys with physiological and behavioral characteristics closer to humans in future studies as a supplement to the glaucoma model, which will help us further study the pathogenesis and treatment of glaucoma and enhance the depth and breadth of the research. (3) Model validation: Before-and-after comparison of retinal function (ERG, PERG) and vision (visual acuity, e.g., qOMR) is missing. In the future, when the experimental conditions are met, we will supplement and at the same time conduct a more convincing verification of the specific staining of the retinal ganglion.

## 6. Conclusions

Optic nerve dysfunction can be successfully generated in mice by injecting photocrosslinkable sericin hydrogel into their anterior chambers, simulating chronic ocular hypertension. The model demonstrated a clear effect of lowering IOP after treatment with tafluprost and timolol maleate eye drops, and the IOP tended to rise after drug withdrawal, demonstrating that the model was very sensitive to medications. The model had some degree of safety, as there was essentially no impact on cell proliferation when sericin toxicity was detected using MTS on cells. In summary, anterior chamber injection of photocrosslinkable sericin hydrogel can provide a stable, effective, and safe model of chronic intraocular hypertension and provide a certain guarantee for the basic research of glaucoma.

## Data Availability

Source data are available from the corresponding author upon reasonable request.

## References

[1] Tham Y.-C., Li X., Wong T.Y., Quigley H.A., Aung T., Cheng C.-Y. (2014). Global Prevalence of Glaucoma and Projections of Glaucoma Burden through 2040: A Systematic Review and Meta-Analysis. Ophthalmology.

[2] Calkins D.J. (2012). Critical Pathogenic Events Underlying Progression of Neurodegeneration in Glaucoma. Prog. Retin. Eye Res..

[3] Bouhenni R.A., Dunmire J., Sewell A., Edward D.P. (2012). Animal Models of Glaucoma. J. Biomed. Biotechnol..

[4] Cone F.E., Gelman S.E., Son J.L., Pease M.E., Quigley H.A. (2010). Differential Susceptibility to Experimental Glaucoma among 3 Mouse Strains Using Bead and Viscoelastic Injection. Exp. Eye Res..

[5] Zhang J., Li L., Huang H., Fang F., Webber H.C., Zhuang P., Liu L., Dalal R., Tang P.H., Mahajan V.B. (2019). Silicone Oil-Induced Ocular Hypertension and Glaucomatous Neurodegeneration in Mouse. eLife.

[6] Fu C.T., Sretavan D. (2010). Laser-Induced Ocular Hypertension in Albino CD-1 Mice. Investig. Ophthalmol. Vis. Sci..

[7] Morrison J.C. (2005). Elevated Intraocular Pressure and Optic Nerve Injury Models in the Rat. J. Glaucoma.

[8] Vecino E., Urcola H., Bayon A., Sharma S.C. (2018). Ocular Hypertension/Glaucoma in Minipigs: Episcleral Veins Cauterization and Microbead Occlusion Methods. Methods Mol. Biol..

[9] Pang I.-H., Clark A.F. (2020). Inducible Rodent Models of Glaucoma. Prog. Retin. Eye Res..

[10] Razali N., Agarwal R., Agarwal P., Kapitonova M.Y., Kannan Kutty M., Smirnov A., Salmah Bakar N., Ismail N.M. (2015). Anterior and Posterior Segment Changes in Rat Eyes with Chronic Steroid Administration and Their Responsiveness to Antiglaucoma Drugs. Eur. J. Pharmacol..

[11] Levkovitch-Verbin H. (2020). Optic Nerve Cupping Represents Neuronal Loss. Ophthalmology.

[12] Lafuente M.P., Villegas-Pérez M.P., Sobrado-Calvo P., García-Avilés A., Miralles de Imperial J., Vidal-Sanz M. (2001). Neuroprotective Effects of Alpha(2)-Selective Adrenergic Agonists against Ischemia-Induced Retinal Ganglion Cell Death. Investig. Ophthalmol. Vis. Sci..

[13] Wamsley S., Gabelt B.T., Dahl D.B., Case G.L., Sherwood R.W., May C.A., Hernandez M.R., Kaufman P.L. (2005). Vitreous Glutamate Concentration and Axon Loss in Monkeys with Experimental Glaucoma. Arch. Ophthalmol..

[14] Zhu W., Zhou Z., Huang Y., Liu H., He N., Zhu X., Han X., Zhou D., Duan X., Chen X. (2023). A Versatile 3D-Printable Hydrogel for Antichondrosarcoma, Antibacterial, and Tissue Repair. J. Mater. Sci. Technol..

[15] Tan D., Zhu W., Liu L., Pan Y., Xu Y., Huang Q., Li L., Rao L. (2023). In Situ Formed Scaffold with Royal Jelly-Derived Extracellular Vesicles for Wound Healing. Theranostics.

[16] Pantcheva M.B., Seibold L.K., Awadallah N.S., Kahook M.Y. (2011). Tafluprost: A Novel Prostaglandin Analog for Treatment of Glaucoma. Adv. Ther..

[17] Metheetrairut A., Leumsamran P., Rojananin S., Kitnarong N. (2012). A Comparison of 0.1% Timolol Eye Gel and 0.5% Timolol Eye Drop in Patients with Chronic Angle-Closure Glaucoma. J. Med. Assoc. Thail..

[18] Zhao Y., Zhang F., Pan Z., Luo H., Liu K., Duan X. (2019). LncRNA NR_003923 Promotes Cell Proliferation, Migration, Fibrosis, and Autophagy via the miR-760/miR-215-3p/IL22RA1 Axis in Human Tenon’s Capsule Fibroblasts. Cell Death Dis..

[19] Kim H.-G., Park J.-W., Park S.-W. (2013). Experimental Chronic Ocular Hypertension by Anterior Chamber Injection of 0.3% Carbomer Solution in the Rat. Clin. Exp. Ophthalmol..

[20] Aragón-Navas A., Rodrigo M.J., Garcia-Herranz D., Martinez T., Subias M., Mendez S., Ruberte J., Pampalona J., Bravo-Osuna I., Garcia-Feijoo J. (2022). Mimicking Chronic Glaucoma over 6 Months with a Single Intracameral Injection of Dexamethasone/Fibronectin-Loaded PLGA Microspheres. Drug Deliv..

[21] Zhong N., Scearce-Levie K., Ramaswamy G., Weisgraber K.H. (2008). Apolipoprotein E4 Domain Interaction: Synaptic and Cognitive Deficits in Mice. Alzheimers Dement. J. Alzheimers Assoc..

[22] Zeng H., Liu N., Yang Y.-Y., Xing H.-Y., Liu X.-X., Li F., La G.-Y., Huang M.-J., Zhou M.-W. (2019). Lentivirus-Mediated Downregulation of α-Synuclein Reduces Neuroinflammation and Promotes Functional Recovery in Rats with Spinal Cord Injury. J. Neuroinflammation.

[23] Lee Y., Morrison B.M., Li Y., Lengacher S., Farah M.H., Hoffman P.N., Liu Y., Tsingalia A., Jin L., Zhang P.-W. (2012). Oligodendroglia Metabolically Support Axons and Contribute to Neurodegeneration. Nature.

[24] Mao X.W., Byrum S., Nishiyama N.C., Pecaut M.J., Sridharan V., Boerma M., Tackett A.J., Shiba D., Shirakawa M., Takahashi S. (2018). Impact of Spaceflight and Artificial Gravity on the Mouse Retina: Biochemical and Proteomic Analysis. Int. J. Mol. Sci..

[25] Naguib S., Backstrom J.R., Gil M., Calkins D.J., Rex T.S. (2021). Retinal Oxidative Stress Activates the NRF2/ARE Pathway: An Early Endogenous Protective Response to Ocular Hypertension. Redox Biol..

[26] Howell G.R., Libby R.T., Jakobs T.C., Smith R.S., Phalan F.C., Barter J.W., Barbay J.M., Marchant J.K., Mahesh N., Porciatti V. (2007). Axons of Retinal Ganglion Cells Are Insulted in the Optic Nerve Early in DBA/2J Glaucoma. J. Cell Biol..

[27] Lin B., Wang S.W., Masland R.H. (2004). Retinal Ganglion Cell Type, Size, and Spacing Can Be Specified Independent of Homotypic Dendritic Contacts. Neuron.

[28] Tribble J.R., Otmani A., Kokkali E., Lardner E., Morgan J.E., Williams P.A. (2021). Retinal Ganglion Cell Degeneration in a Rat Magnetic Bead Model of Ocular Hypertensive Glaucoma. Transl. Vis. Sci. Technol..

[29] Chung K., Wallace J., Kim S.-Y., Kalyanasundaram S., Andalman A.S., Davidson T.J., Mirzabekov J.J., Zalocusky K.A., Mattis J., Denisin A.K. (2013). Structural and Molecular Interrogation of Intact Biological Systems. Nature.

[30] Chen H., Wei X., Cho K.-S., Chen G., Sappington R., Calkins D.J., Chen D.F. (2011). Optic Neuropathy Due to Microbead-Induced Elevated Intraocular Pressure in the Mouse. Investig. Ophthalmol. Vis. Sci..

[31] Yu H., Zhong H., Chen J., Sun J., Huang P., Xu X., Huang S., Zhong Y. (2020). Efficacy, Drug Sensitivity, and Safety of a Chronic Ocular Hypertension Rat Model Established Using a Single Intracameral Injection of Hydrogel into the Anterior Chamber. Med. Sci. Monit. Int. Med. J. Exp. Clin. Res..

[32] Nadal-Nicolás F.M., Galindo-Romero C., Lucas-Ruiz F., Marsh-Amstrong N., Li W., Vidal-Sanz M., Agudo-Barriuso M. (2023). Pan-Retinal Ganglion Cell Markers in Mice, Rats, and Rhesus Macaques. Zool. Res..

[33] Nadal-Nicolás F.M., Sobrado-Calvo P., Jiménez-López M., Vidal-Sanz M., Agudo-Barriuso M. (2015). Long-Term Effect of Optic Nerve Axotomy on the Retinal Ganglion Cell Layer. Investig. Ophthalmol. Vis. Sci..

[34] Zhang L., Li G., Shi M., Liu H.-H., Ge S., Ou Y., Flanagan J.G., Chen L. (2017). Establishment and Characterization of an Acute Model of Ocular Hypertension by Laser-Induced Occlusion of Episcleral Veins. Investig. Ophthalmol. Vis. Sci..

[35] Patil S.V., Kasetti R.B., Millar J.C., Zode G.S. (2022). A Novel Mouse Model of TGFβ2-Induced Ocular Hypertension Using Lentiviral Gene Delivery. Int. J. Mol. Sci..

[36] Zahavi A., Friedman Gohas M., Sternfeld A., Daoud Zreiq N., Muhsinoglu O., Ofri R., BarKana Y., Goldenberg-Cohen N. (2022). Histological and Molecular Characterization of Glaucoma Model Induced by One or Two Injections of Microbeads to the Anterior Chamber of Mice. Int. Ophthalmol..

[37] Zhu H., Wu X., Liu R., Zhao Y., Sun L. (2023). ECM-Inspired Hydrogels with ADSCs Encapsulation for Rheumatoid Arthritis Treatment. Adv. Sci..

[38] Jian X., Feng X., Luo Y., Li F., Tan J., Yin Y., Liu Y. (2021). Development, Preparation, and Biomedical Applications of DNA-Based Hydrogels. Front. Bioeng. Biotechnol..

[39] Ma S., Gu S., Zhang J., Qi W., Lin Z., Zhai W., Zhan J., Li Q., Cai Y., Lu Y. (2022). Robust Drug Bioavailability and Safety for Rheumatoid Arthritis Therapy Using D-Amino Acids-Based Supramolecular Hydrogels. Mater. Today Bio.

[40] Wu K.Y., Ashkar S., Jain S., Marchand M., Tran S.D. (2023). Breaking Barriers in Eye Treatment: Polymeric Nano-Based Drug-Delivery System for Anterior Segment Diseases and Glaucoma. Polymers.

[41] Huang W., Wang L., Yang R., Hu R., Zheng Q., Zan X. (2022). Combined Delivery of Small Molecule and Protein Drugs as Synergistic Therapeutics for Treating Corneal Neovascularization by a One-Pot Coassembly Strategy. Mater. Today Bio.

[42] Park S.-W., Lee J., Kook M.S. (2022). Efficacy, Safety and Patient-Reported Outcomes with Preservative-Free (PF) Tafluprost or PF-Dorzolamide/Timolol Compared with Preserved Latanoprost: A Prospective Multicenter Study in Korean Glaucoma Patients with Ocular Surface Disease. Pharmaceuticals.

[43] Yang Q., Cho K.-S., Chen H., Yu D., Wang W.-H., Luo G., Pang I.-H., Guo W., Chen D.F. (2012). Microbead-Induced Ocular Hypertensive Mouse Model for Screening and Testing of Aqueous Production Suppressants for Glaucoma. Investig. Ophthalmol. Vis. Sci..

[44] Poursamar S.A., Lehner A.N., Azami M., Ebrahimi-Barough S., Samadikuchaksaraei A., Antunes A.P.M. (2016). The Effects of Crosslinkers on Physical, Mechanical, and Cytotoxic Properties of Gelatin Sponge Prepared via in-Situ Gas Foaming Method as a Tissue Engineering Scaffold. Mater. Sci. Eng. C Mater. Biol. Appl..

